# Increased oxidative stress and severe arterial remodeling induced by permanent high-flow challenge in experimental pulmonary hypertension

**DOI:** 10.1186/1465-9921-12-119

**Published:** 2011-09-09

**Authors:** Peter Dorfmüller, Marie-Camille Chaumais, Maria Giannakouli, Ingrid Durand-Gasselin, Nicolas Raymond, Elie Fadel, Olaf Mercier, Frédéric Charlotte, David Montani, Gérald Simonneau, Marc Humbert, Frédéric Perros

**Affiliations:** 1 Université Paris-Sud, Faculté de médecine, Kremlin-Bicêtre, F-94276, France; 2 AP-HP, Centre National de Référence de l'Hypertension Pulmonaire Sévère, Service de Pneumologie et Réanimation Respiratoire, Hôpital Antoine Béclère, Clamart, France; 3 INSERM U999, Hypertension Artérielle Pulmonaire: Physiopathologie et Innovation Thérapeutique, Clamart - Le Plessis-Robinson, France; 4 Service d'Anatomie et Cytologie Pathologiques, Hôpital Marie Lannelongue, Le Plessis Robinson, France; 5 Service d'Anatomie et de Cytologie Pathologiques, Groupe Hospitalier Pitié-Salpêtrière, Université Pierre et Marie Curie, Paris, France

## Abstract

**Background:**

Involvement of inflammation in pulmonary hypertension (PH) has previously been demonstrated and recently, immune-modulating dendritic cells (DCs) infiltrating arterial lesions in patients suffering from idiopathic pulmonary arterial hypertension (IPAH) and in experimental monocrotaline-induced PH have been reported. Occurrence of perivascular inflammatory cells could be linked to local increase of oxidative stress (OS), as it has been shown for systemic atherosclerosis. The impact of OS on vascular remodeling in PH is still to be determined. We hypothesized, that augmented blood-flow could increase OS and might thereby contribute to DC/inflammatory cell-recruitment and smooth-muscle-cell-proliferation.

**Methods:**

We applied a monocrotaline-induced PH-model and combined it with permanent flow-challenge. Thirty Sprague-Dawley rats were assigned to following groups: control, monocrotaline-exposure (MCT), monocrotaline-exposure/pneumonectomy (MCT/PE).

**Results:**

Hemodynamic exploration demonstrated most severe effects in MCT/PE, corresponding in histology to exuberant medial and adventitial remodeling of pulmonary muscular arteries, and intimal remodeling of smaller arterioles; lung-tissue PCR evidenced increased expression of DCs-specific fascin, CD68, proinflammatory cytokines (IL-6, RANTES, fractalkine) in MCT/PE and to a lesser extent in MCT. Major OS enzyme NOX-4 was maximal in MCT/PE. Antioxidative stress enzymes Mn-SOD and glutathion-peroxidase-1 were significantly elevated, while HO-1 showed maximal expression in MCT with significant decrease in MCT/PE. Catalase was decreased in MCT and MCT/PE. Expression of NOX-4, but also of MN-SOD in MCT/PE was mainly attributed to a highly increased number of interstitial and perivascular CXCR4/SDF1 pathway-recruited mast-cells. Stress markers malonedialdehyde and nitrotyrosine were produced in endothelial cells, medial smooth muscle and perivascular leucocytes of hypertensive vasculature. Immunolabeling for OX62, CD68 and actin revealed adventitial and medial DC- and monocyte-infiltration; in MCT/PE, medial smooth muscle cells were admixed with CD68^+^/vimentin^+ ^cells.

**Conclusion:**

Our experimental findings support a new concept of immunologic responses to increased OS in MCT/PE-induced PAH, possibly linking recruitment of dendritic cells and OS-producing mast-cells to characteristic vasculopathy.

## Background

Pulmonary arterial hypertension (PAH) is a fatal condition that may be without obvious cause or complicating a limited spectrum of conditions [1]. The previous determination of responsible gene mutations, concerning bone morphogenetic protein receptor II (BMPRII) [2], activin receptor-like kinase-1 (ALK-1) [3] and recent discovery of dysregulated genes encoding for hypoxia inducible factor 3-alpha (HIF-3 alpha), mitochondrial cytochrome-c oxidase, and superoxide dismutase-2 in human and experimental PAH [4] provide a new understanding of genetic predisposition to PAH. However, additional biologic abnormalities and histologic features, such as inflammation, appear to link different forms of PAH. Indeed, PAH is a common complication of systemic inflammatory conditions, including scleroderma or systemic lupus erythematosus, and pulmonary arterial lesions in the lungs of patients suffering from connective tissue diseases with isolated PAH are often similar to those found in lungs displaying idiopathic PAH (IPAH) [5]. Inflammatory cell infiltrates in the range of affected pulmonary arteries, including macrophages and lymphocytes, have been reported in IPAH and PAH associated with other conditions [5-7]. In addition our group has previously provided evidence for the recruitment of monocyte-derived immune-modulating cells, so called dendritic cells, into pulmonary arterial lesions of both human PAH and monocrotaline-induced pulmonary hypertension in rats [8]. Of note, increased plasma concentrations of proinflammatory cytokines IL-1 and IL-6 and elevated expression of chemokines RANTES and fractalkine in endothelial cells of affected pulmonary arteries have been demonstrated in PAH [9-11]. The relation of immune responses and inflammatory elements to the mediation of mitogenic activity through growth factors initiating endothelial- and smooth-muscle proliferation is of particular interest in the setting of PAH. We have previously shown that fractalkine, a chemokine expressed in endothelial cells of altered pulmonary arteries in patients displaying PAH, not only exerts chemoattraction on T-lymphocytes and thereby contributes to perivascular inflammation, but additionally induces smooth muscle cell proliferation [9,8]. Moreover we have recently reported increased expression of PDGF-B and -A in the arterial wall of PAH-lungs (smooth-muscle cells and endothelial cells) and presence of its receptors PDGFR-beta and -alpha on medial smooth muscle cells, suggesting a crosstalk between the intimal and the medial arterial layer, leading to concerted vascular compartment reactions and finally to occlusive remodeling [12].

Oxidative stress (OS) is accepted as a pivotal player in the pathophysiology of vascular diseases and has been identified as an important trigger of inflammatory processes within the vascular wall, especially in the setting of systemic arterial disease [13]. Enhanced superoxide (·O2^-^) production increases nitric oxide (NO) inactivation, which constitutes the main vascular vasodilator. Dismutation of superoxide anions and reaction of NO with ·O2^- ^leads to an accumulation of hydrogen peroxide and peroxynitrites, which will cause cell damage [14,15]. Linkage of reactive oxygen species production and expression to chemotactic and mitogenic factors such as cyclophyllin A has been recently described for atheromatous lesions and aortic aneurysm [16]. Satoh and co-workers have shown that this pro-inflammatory chaperone protein which is secreted from smooth muscle cells of systemic arteries in response to ROS is responsible for intimal and medial remodeling, emphasizing the association of OS, pro-inflammatory conditions and vascular remodeling. In systemic arteries, DCs accumulate in regions displaying hemodynamic stress by turbulent flow conditions and being prone to hypertensive intimal lesions [17]. A possible link between DCs and OS in hypertensive diseases is provided by observations relating increased pulsatile flow conditions to pathologic generation of ROS in cultured endothelial cells [18]. Furthermore, Zhu and coworkers have recently presented experimental data suggesting that homocysteine-stimulated endothelial cells strikingly increase ROS generation together with an augmented DC adhesion and transmigration, while NO release is markedly decreased [19]. Vice versa, pretreatment with antioxidant before homocysteine-stimulation markedly attenuated the induction of DC adhesion and transmigration, dependent on the intracellular ROS decrease and endothelial NO increase. Finally, recruitment of macrophages and lymphocytes to pulmonary arteries in PAH has recently been related to increase of OS products, such as nitrotyrosine, or 8-hydroxyguanosine [20].

On the basis of our observations on recruited DCs in PAH, we hypothesized that in an high blood-flow triggered stress model of PH, the increase in ROS could have impact on cell proliferation in typical vascular lesions and recruited cells, including DCs.

## Methods

### Animal model and study design

Twelve week-old male, 350- to 400 g, pathogen-free Sprague-Dawley rats were studied. Rats were randomly assigned to one of three groups. The control group (C) (*n *= 10 at study day 40) was created by a single subcutaneous injection of saline on study-day 0. Group MCT received a single subcutaneous monocrotaline-treatment on day 0 (*n *= 10 at study day 40). Group MCT/PE received a single subcutaneous monocrotaline-treatment on day 0 and subsequently underwent left pneumonectomy on day 7 (*n *= 10 at study day 40: due to increased peri-operative mortality of the MCT/PE group, sufficient animals were submitted to pneumonectomy in order to reach a final group number of 10).

### Operative procedures

Monocrotaline (Sigma Aldrich^®^) was dissolved in 1 N HCL and pH was adjusted to 7.40 with 1 N NaOH. A dose of 60 mg/kg was administered to 20 animals on day 0, by subcutaneous injection into the cervical region. One week after monocrotaline injection, left pneumonectomy was performed on 10 individuals. Rats were anesthesized by intraperitoneal injection of ketamine (35 mg/kg), xylasine (4 mg/kg) and acepromazine (0.5 mg/kg). Rats were then surgically tracheotomized under aseptic conditions and intubation was performed with a Cathlon^® ^16G by ventilating 2 ml of ambient air, at a frequency of 60/minute, with PEEP at 1.0 cm H2O. After shaving the operation site and placing rats in a half-supine position, thoracotomy and left pneumonectomy were performed with aseptic technique. Eventually, thoracotomy was closed by ligature of muscular and dermal layers, after positioning of a trans-thoracic drain-tube. The latter was withdrawn after creation of a negative intrapleural pressure of -20 mmHg. Finally, rats were extubated and tracheotomy was closed. All animals received appropriate care in accordance with the "Principles of Laboratory Animal Care" formulated by the National Society for Medical Research and the Guide for the Care of Laboratory Animals prepared by the National Academy of Science and published by the National Institutes of Health (NIH 86-23, revised 1985). The study was approved by the administrative panel on animal care from Centre de Chirurgie Expérimentale Marie Lannelongue, Le Plessis-Robinson, and was conducted according to South Paris University regulations.

### Hemodynamic studies

On day 40, after final weighing, right ventricular pressure, systolic, diastolic and mean pulmonary arterial pressures were recorded. Rats were anesthesized as described above. Right-heart catheterization was performed as previously described ^18^. An umbilical catheter (external diameter = 1.2 mm; Tyco^®^, Plaisir, France) was heat-formed to achieve an ideal 90° angle on the distal extremity (1 cm) and a slightly curved tip. The catheter was connected to a recording apparatus, consisting of a pressure-head (Ohmeda^®^, Trappes, France), an amplifier (CGR^®^, St. Cloud, France) and a recorder (Sefram^®^, St. Etienne, France). The jugular vein was exposed by blunt dissection. The catheter was inserted and positioned 2.5 to 3 cm from sting, corresponding to right intra-auricular position. By rotating the catheter a right intra-ventricular position was achieved and further insertion from this point allowed access to the pulmonary artery, supported by the curved catheter-form. Throughout this operation, catheter position was controlled by pressure waveform monitoring of atrium, ventricle and pulmonary artery.

### Tissue preparation

After undergoing hemodynamic measurements, all 30 rats were sacrificed by bleeding through aortal dissection. Post-mortem explanted lungs were distended by intra-tracheal infusion of OCT compound (VWR^®^), diluted in phosphate buffered saline (PBS) (1:1), to preserve lung morphology. After quickly freezing in isopentane on dry ice, lungs were stored at -80°C until further processing.

Explanted hearts of all rats underwent quantitative morphometry. The right ventricle (RV) was dissected from left ventricle (LV) and septum (S). Then, RV and LV+S were weighed and the following ratio was calculated to estimate right ventricle hypertrophy: [RV_weight_]/[(LV+S)_weight_].

### mRNA quantification by real-time reverse transcription polymerase chain reaction (RT-PCR)

For gene expression quantification by real-time reverse transcription polymerase chain reaction we used Applied Biosystems TaqMan Gene Expression Assays with TaqMan Universal PCR Master Mix and reactions were run in an ABI Prism 7000 Sequence Detection System (Applied Biosystems). Results were analyzed with the second derivative maximum method to set CT with 18s as an internal housekeeping gene control. The following gene expression assays were used: Fascin (Rn01452400_m1), IL-6 (Rn00561420_m1), CX3CL1 (Rn00593186_m1), RANTES (Rn00579590_m1), CD68 (Rn01495632_m1), HO-1 (Rn00561387_m1), NOX-1 (Rn00586652_m1), NOX-2 (Rn00576710_m1), NOX-3 (Rn01430441_m1), NOX-4 (Rn00585380_m1), Mn-SOD (Rn00690587_g1), catalase (Rn00560930_m1), Glutathione peroxidase-1 (Rn00577994_g1), and ribosomal 18s (Hs99999901_s1), and tryptase (Rn00570928_m1)

### Histochemistry

The right lung from all animals was processed for histomorphologic evaluation. Three frozen lung sections (7 μm) from lower, medial and upper lobe of each animal were stained with erythrosine (RAL^®^) and then counterstained with Mayer's hematoxyline (Labonord^®^). Evaluation was performed by two experienced pathologists (PD, FC).

### Immunohistochemistry

Immunohistochemical experiments were performed on 7 μm-thick sections of frozen lung tissue. After routine preparation, rat samples were processed with the following antibodies: Rat dendritic cells were stained with the monoclonal anti-CD103 antibody (Pharmingen^®^, clone OX-62) diluted in PBS containing 2% normal rat serum. Rat OX-62 labeling was revealed with the kit LSAB 2 for use on rat specimens (Dako^®^). Macrophages were detected with anti-CD68 (Serotec, clone ED1), Mast cells with anti-Tryptase (Sigma, WH0007177M1) Pro-inflammatory cytokines were evaluated after staining with antibodies against IL-6 (Abcam, ab6672), RANTES (Torrey Pines, TP211), fractalkine (Torrey Pines TP203), and SDF-1 (R&D systems, MAB350). OS markers were revealed by antibodies against NOX-4 (Epitomics, 3174-1), MN-SOD (Epitomics, 2299-1), malonedialdehyde (Abcam, ab6463) and nitrotyrosine (Santa Cruz, (39B6) sc-32757). Staining was completed after incubation with substrate-chromogen AEC solution (Dako^®^). Slides were counterstained with Mayer's Hematoxyline (Labonord ^®^) and mounted with aqueous medium (Glycergel, Dako^®^). Controls used for these antibodies included omission of the primary antibody and substitution of the primary antibody by isotype control.

### Immunofluorescent labeling

For double immunofluorescence, anti-rat anti-OX-62-(Pharmingen), or anti-NOX-4 (Epitomics, 3174-1) antibodies were labeled by Biogenex's or Dako's biotinylated anti-mouse immunoglobulins after overnight incubation at 4°C and streptavidin, Alexa Fluor 594 or 488 conjugate (Molecular Probes) for one hour. Then, the tissues were incubated overnight either with FITC conjugated, anti-smooth muscle alpha-actin (Sigma-aldrich clone 1A4), anti-CXCR4 (R&D systems MAB172), rhodamine conjugated vimentine (Santa Cruz sc-6260), or Alexa Fluor 488 conjugated anti-rat CD68 (Serotec clone ED1). The slides were mounted with Vectashield Mounting Medium with DAPI (Vector Laboratories).

### Statistical analysis

All data are given as mean ± SEM. Analysis of variance (ANOVA) using repeated measures and the Fisher projected least significant difference (PLSD) post test was performed on the results. A p value of less than 0.05 was considered significant.

## Results

Thirty rats were studied. Ten received saline subcutaneous injection, 20 received monocrotaline subcutaneous injection (60 mg/kg) and 10 out of these 20 underwent left pneumonectomy. Due to increased peri-operative mortality of the MCT/PE group, we submitted a sufficient number of animals to pneumonectomy in order to reach a group number of 10 on study-day 40. Hemodynamic data were obtained shortly before sacrifice on day 40. All morphometry-, RT-PCR-, immunohistochemistry-, and immunofluorescence-experiments were performed after sacrifice (day 40).

### Hemodynamics and quantitative morphometry

**Figure 1:**
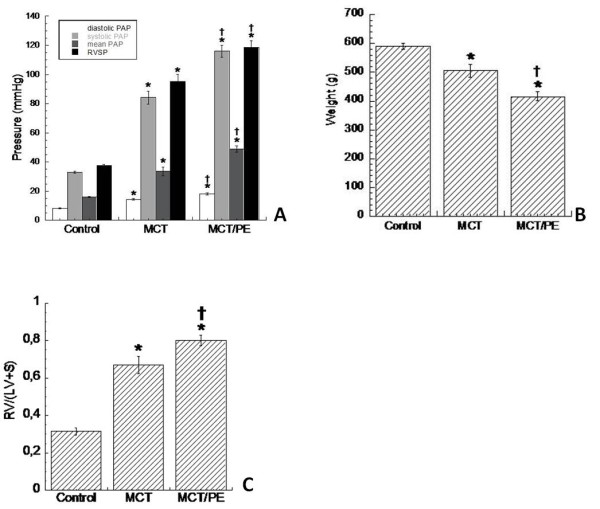
**Hemodynamics, weight, and right ventricular hypertrophy in experimental PH models and controls**. **A**: diastolic, systolic, and mean PAP, as well as RVSP (in mmHg) for the different animal-groups. **B**: body weight (in g), obtained at day 40, shortly before catheterization and sacrifice. **C**: right ventricular hypertrophy (ratio) calculated as follows: [RV_weight_]/[(LV+S)_weight_]. * P < 0.05 versus control; † P < 0.05 versus MCT.

Monocrotaline-challenged rats (MCT) developed severe pulmonary hypertension within 40 days. In consequence, right ventricular systolic pressure (RVSP) and mean pulmonary artery pressure (mPAP) was increased significantly as compared with the control group (subcutaneous saline injection) (Figure 1A). Body weight of both treated groups significantly decreased, as compared to controls, and MCT-PE rats had significant decrease in body weight, as compared to MCT-rats (Figure 1B). A significant RV hypertrophy developed in MCT as a consequence of increased pulmonary resistances. The ratio [RV_weight_]/[(LV+S)_weight_] increased from 0.3 ± 0.03 in C, to 0.81 ± 0.04 in MCT, after 40 days in both groups. All analyzed animals survived until day 40. Monocrotaline-treated rats with pneumonectomy (MCT/PE) had the highest values for RVSP and mPAP. [RV_weight_]/[(LV+S)_weight_] ratio was 0.95 ± 0.04 on day 40 (Figure 1C).

### Microscopic morphometry

**Figure 2:**
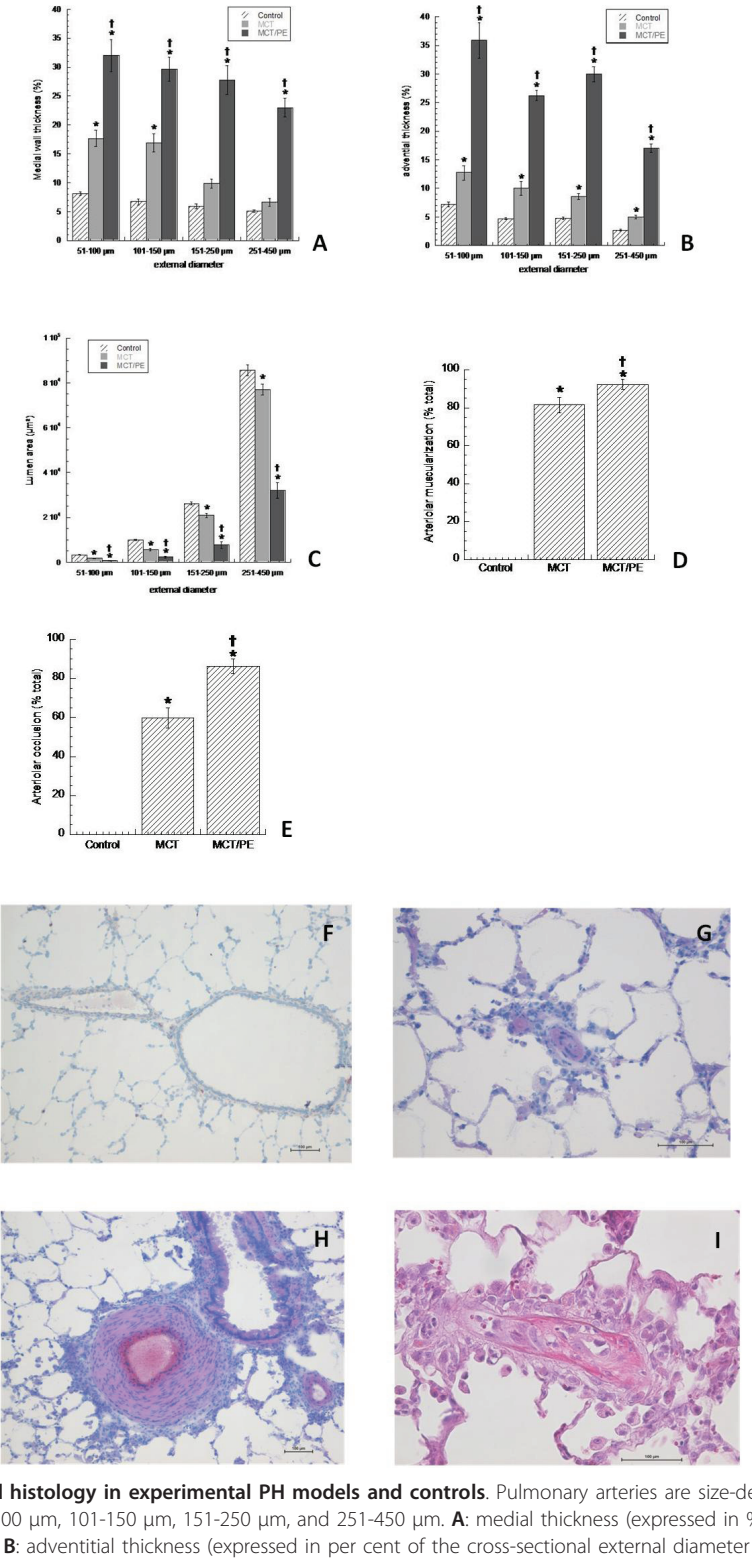
**Morphometry and histology in experimental PH models and controls**. Pulmonary arteries are size-dependently differentiated into the following categories: 51-100 μm, 101-150 μm, 151-250 μm, and 251-450 μm. **A**: medial thickness (expressed in % of the cross-sectional external diameter of arteries). **B**: adventitial thickness (expressed in per cent of the cross-sectional external diameter of arteries). **C**: lumen area (in μm^2^). **D**: arteriolar muscularization (in % of total arteriolar number). **E**: virtual arteriolar occlusion (loss of visually perceptible lumen, in % of total arteriolar number). **F**: normal pulmonary artery (PA) (control) with adjacent bronchiole (HE staining). **G**: muscularized, virtually occluded arteriole in a MCT/PE-animal (HE-staining). **H**: excessive medial thickening in a large PA of an animal from the MCT/PE-group (HE-staining). **I**: intimal thickening of a pulmonary arteriole in a MCT/PE animal (HE-staining). * P < 0.05 versus control; † P < 0.05 versus MCT (Figures 2A-E).

Lung samples of all animals were evaluated on HE- and actin-stained slides. Medial wall thickness of pulmonary arteries (PA)/arterioles between 51-100 μm, as well as PA between 101-150 μm, 151-250 μm and 251-450 μm were determined separately. Medial thickness increased significantly in MCT-animals as compared to the control group. The most important changes were found in PA of 51-150 μm size, while PA of 151-450 μm displayed moderate changes (Figure 2A). MCT/PE-animals consistently presented the highest values for medial thickness. PA of 151-250 μm, and to a lesser extent of 251-450 μm, showed intense medial remodeling. We measured adventitial thickness for all groups and correlated them with the external arterial diameter (Figure 2B). We found constant increase of adventitial diameter in treated groups with significant difference between all. Lumen areas decreased for MCT and to a higher degree for MCT/PE, with significant changes for both groups in PA of all size (Figure 2C). We quantitatively assessed the degree of muscularization of pulmonary arterioles with a diameter between 25 and 50 μm, counting 10 arterioles of this size for each slide, at random (Figure 2D). Control-animals showed virtually no muscularized arterioles of this size. In MCT, the majority of counted arteries were clearly muscularized, with a circumferential, actin positive cell layer. In animals of the MCT/PE group, the percentage of muscularized arterioles was close to the entire number of counted arterioles. This pseudo-medial muscularization could not be quantified in terms of thickness, due to the lacking characteristics of the tunica media in arteries, that is to say well-defined internal and external elasticae. Complete occlusion of arterioles was separately assessed (Figure 2E) and corresponded by trend to the observations made in Figure 2D. In MCT/PE the vast majority of muscularized arterioles appeared occluded.

Examples of a normal pulmonary artery (control) and of remodeled pulmonary arteries and arterioles (MCT/PE) are shown in Figures 2F-H. In addition, arterioles of the MCT-PE group partially displayed intimal thickening corresponding to collagen-rich fibrosis, a feature that was never observed in the MCT group (Figure 2I).

### RT-PCR

#### Expression of DCs, macrophages and mast cells

**Figure 3:**
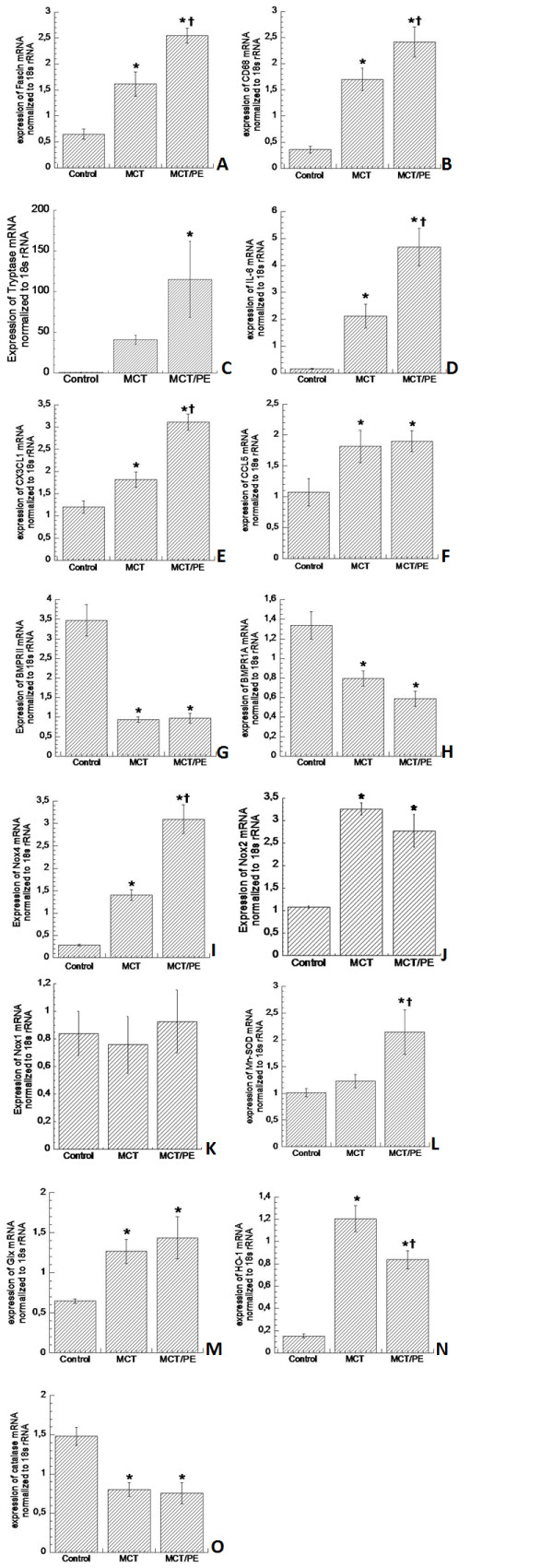
**mRNA gene expression measured by real-time PCR**. 18s mRNA was used as an internal housekeeping control. Results are provided as arbitrary units. **A**: Fascin. **B**: CD68. **C**: tryptase. **D**: Interleukin-6. **E**: CX3CL1 (fractalkine). **F**: CCL5 (RANTES). **G**: BMPR2. **H**: BMPR1A. **I**: NOX-4. **J**: NOX-2. **K**: NOX-1. **L**: Mn-SOD. **M**: Glutathione peroxidase-1. **N**: Heme oxygenase-1. **O**: Catalase. * P < 0.05 versus control; † P < 0.05 versus MCT.

DC-specific fascin gene expression was increased in animals of the MCT- and the MCT/PE-group, with 2.4-fold and 3.7 fold higher mRNA-expressions, as compared to control animals, respectively (Figure 3A). Increase of macrophage-specific CD68 gene expression in MCT and MCT/PE animals had a similar order of magnitude (Figure 3B). Mast cell-specific tryptase was most dramatically increased in both models as compared to control rats, with a 110-fold elevation in MCT/PE, and a 40-fold increase in MCT (Figure 3C).

#### Cytokine-expression

Proinflammatory cytokines were evaluated: IL-6 expression showed marked elevation with gradual increase from MCT to MCT/PE. Animals of the latter group had a 5-fold IL-6 expression increase as compared to C (Figure 3D). Chemokines fractalkine and RANTES exhibited significantly increased expression in lungs of hypertensive rats, with significant higher values for fractalkine in MCT/PE, as compared to MCT (Figure 3E, F).

#### PAH-associated genes

BMPR1 and BMPR2 were significantly reduced in hypertensive animals with a 4-fold decrease for BMPR2 in both, MCT- and MCT/PE-animals (Figure 3G). BMPR1-decrease was more pronounced in MCT/PE as compared to MCT, although it did not reach statistical significance (Figure 3H).

#### Oxidant and anti-oxidant enzymes

Testing of major oxidant enzyme NOX-4 revealed highest expression in MCT/PE animals (10.3-fold) with significant difference to the MCT-group (4.7-fold) (Figure 3I). NOX-2 displayed a significantly higher expression (3-fold) in the two model groups, with non-significant decrease in the MCT/PE group (Figure 3J). NOX-1 did not show any significant differences between the three groups (Figure 3K), and NOX-3 expression was not detected at all in either group. Anti-oxidant capacities revealed mixed results: as compared to the control group, an increase of anti-oxidants MN-SOD and gluthation-peroxidase (2.2-fold and 2-fold, respectively) was observed in MCT/PE (Figure 3L, M). In animals of the MCT-group only gluthation-peroxidase was significantly increased as compared to controls. Anti-oxidant enzyme HO-1, constituting one of the major defenses against OS and its products (ROS), revealed 7.3-fold increase in MCT as compared to controls. However, animals of the MCT/PE group exhibited 4.8-fold increase as compared to C, or a 1.5-fold decrease as compared to MCT (Figure 3N). Contrary to the general increase in anti-oxidant capacities, anti-oxidant catalase proved significant decrease in both, MCT and MCT/PE (1.8-fold and 2-fold, respectively), as compared to controls (Figure 3O).

### Immunohistochemical analysis

#### Detection of vascular dendritic cells

**Figure 4:**
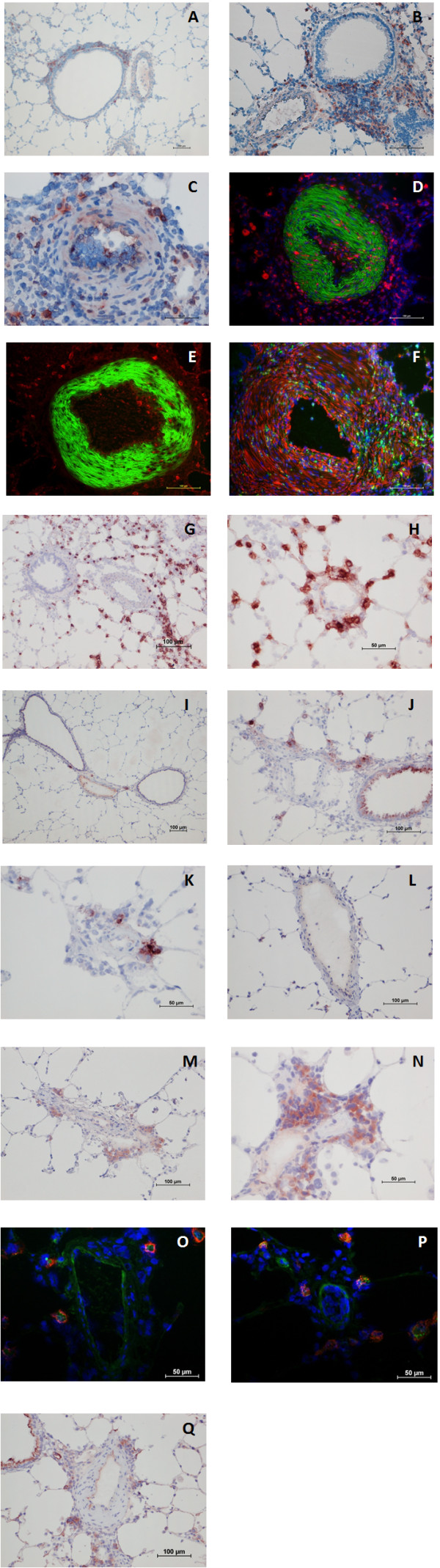
**Immunohistochemical/immunofluorescent findings in lungs from PH models and controls**. **A**: distribution of OX62^+ ^cells in lungs of a control; DCs are detected within BALT. **B**: OX62-staining in MCT-rats; DCs are infiltrating the adventitia and are present in BALT. **C**: PA from a MCT/PE-animal; perivascular OX62+ cells are infiltrating adventitia and media of a remodeled vessel. **D**: double-labeling (anti-CD68/anti-actin) in a PA of a MCT/PE-animal: medial monocytic infiltration. **E**: double-labeling (anti-OX62/anti-actin antibodies in a PA (MCT/PE); DCs infiltrate the media. **F**: double-labeling (anti-CD68/anti-vimentine) in a PA (MCT/PE); cells within the media show co-localization of markers (yellow cells). **G**: anti-NOX-4 staining in a MCT/PE-animal. Interstitial NOX-4+ cells are highlighted. **H**: NOX-4+ cells next to a small arteriole (MCT/PE-animal). **I**: anti-NOX-4 staining in a control. Few cells display positivity. **J**: anti-Mn-SOD staining in a MCT/PE-animal: cells in the range of a remodeled PA are highlighted. **K**: A remodeled arteriole displays positivity for some perivascular cells (MCT/PE-animal). **L**: Control animal showing virtual negativity for Mn-SOD+ cells. **M**: anti-Tryptase staining reveals positive cells (same cytomorphology and distribution) next to a small diseased PA (MCT/PE-animal). **N**: Interstitial tryptase+ cells in the range of an arteriole (MCT/PE-animal). **O**: Immunofluorescent double-staining: merge-photo of a PA in a MCT/PE-animal. NOX-4 = red, CXCR4 = green and Dapi = blue: note NOX-4+/CXCR4+ cells. **P**: Smaller remodeled arteriole displaying cells of the same NOX-4/CXCR4+ phenotype in the perivascular space. **Q**: anti-SDF-1 staining (MCT/PE-animal). Endothelial cells of a diseased PA and a smaller vessel, inflammatory perivascular cells and bronchial epithelial cells are positive.

In all lung samples, OX-62^+ ^dendritic cells were detected. Control-animals showed discrete but constant DCs distribution in bronchiolar-associated lymphatic tissue (BALT), but no involvement within pulmonary arteries (Figure 4A). DCs were also observed in pulmonary septa and pleura of controls. Remodeled pulmonary arteries of MCT-treated animals presented perivascular DCs infiltrating the adventitia, but not medial smooth muscle (Figure 4B). In the MCT/PE group, numerous cells within the intensely remodeled adventitia revealed an OX-62^+ ^phenotype. Here, histiocytes and DCs also infiltrated the media in a transmural manner (Figure 4C-E). In small pulmonary arteries and arterioles, intra-luminal wall-adherent leucocytes were observed, including DCs (Figure 4C). Immunofluorescence double-labeling revealed activated CD68^+^/vimentin^+ ^intramural macrophages (Figure 4F). Staining with NOX-4- and Mn-SOD-antibodies revealed a surprisingly high number of large, cytoplasm-rich cells, containing intracytoplasmic granules (Figure 4G-L). The number of NOX-4+ cells was larger than those being MN-SOD+. The cells were present in the interstitium, in alveolar capillaries and in the vicinity of remodeled pulmonary vessels and bronchioles from MCT/PE animals, and MCT animals. The control group displayed only a few, scattered, NOX-4+/MN-SOD+ cells (Figure 4I, L). Further immunohistochemical and immunofluorescence experiments were engaged in order to identify the cell type: CD68, OX-62, and vimentin did not co-localize with the described cells (not shown). Immunohistochemical staining with anti-Tryptase revealed positivity for the same cytoplasm-rich granulated interstitial and perivascular cells, although antibody specificities did not allow us to perform immunofluorescent double-staining with NOX-4 (Figure 4M, N). Mast-cell attracting pro-inflammatory chemokine SDF-1 and its receptor CXCR4 were tested by immunohistochemistry and immunofluorescence: fluorescent double-staining allowed us to show intense CXCR4-expression by NOX-4+ mast cells, while its ligand SDF-1 was expressed by peri-vascular inflammatory cells and endothelial cells of pulmonary arteries, arterioles and veins, as well as by bronchial epithelial cells (Figure 4O-Q).

#### Localization of ROS-activity and cytokines

**Figure 5:**
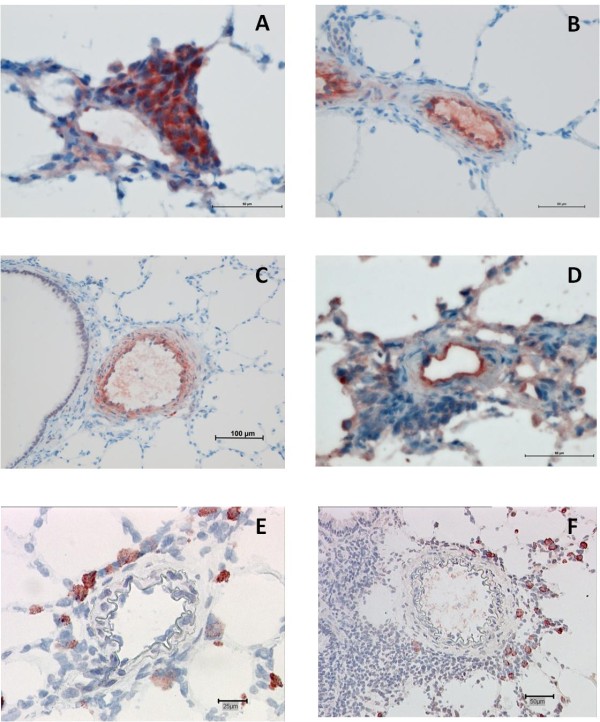
**Immunohistochemical findings in pulmonary arteries and arterioles from experimental PH models**. **A**: pulmonary arteriole of a MCT-treated animal; perivascular cells are staining with anti-malonedialdehyd antibody. **B**: Nitrotyrosin-staining in lungs from a MCT/PE-rat; endothelial cells within a remodeled pulmonary artery show pronounced staining. **C**: Pulmonary artery in an MCT-treated animal: in addition to the endothelial staining, smooth-muscle cells of the tunica media show are, to a lesser extent, nitrotyrosine-positive. **D**: IL-6 staining reveals positive endothelial cells in a small pulmonary artery of MCT/PE-treated animals. **E**: perivascular inflammatory cells express CCL5 (RANTES), **F**: and CX3CL1 (fractalkine) in MCT- and MCT/PE-animals.

Use of anti-malonedialdehyde antibodies evidenced intense endothelial staining of pulmonary arteries/arterioles and veins of MCT- and MCT/PE-animals. Positive staining was also shown for a majority of cells within the BALT and for infiltrating perivascular leucocytes (Figure 5A). Anti-nitrotyrosine incubation led to a positive reaction in endothelial cells of pulmonary vasculature and to a lesser extent in perivascular cells (Figure 5B). Both, nitrotyrosine and to a lesser extent malonedialdehyde were present in smooth muscle cells of affected arteries (Figure 5C). Pulmonary vessels of control-animals showed exclusively discrete positive staining of endothelial cells (not shown). Proinflammatory cytokine IL-6 was detected in endothelial cells of pulmonary arteries, while chemokines RANTES and fractalkine were localized to perivascular leucocytes (Figure 5D-F).

## Discussion

In the present study, we have evaluated the presence of oxidants and anti-oxidants in experimental pulmonary hypertension, their enzymatic source, their impact on pulmonary cellular recruitment and vascular structures. We used a combined inflammatory-/increased flow-model of pulmonary hypertension, first described by Okada and co-workers [21]. The main finding of our study, is that increased flow in MCT-induced pulmonary hypertension potentiates three pathological parameters: production of reactive oxygen species (ROS), remodeling of pulmonary arteries and hemodynamic deterioration. These findings provide new evidence for a vicious circle comprising increased, mast-cell induced OS in the range of high-flow challenged small pulmonary arteries, homing of immune-modulating cells to this "Achille's heel", and their contribution to vascular remodeling through inflammation.

### Mast cell steered OS is increased in PH with additional blood-flow, after pneumonectomy

In our model of combined inflammatory/increased flow-induced PH, we observed significantly elevated expression of pro-oxidative enzyme NOX-4 in lungs of rats from the MCT/PE-group, as compared to controls and to the MCT-group. Pro-oxidative enzyme NOX-2 was significantly increased in both, MCT/PE and MCT. Concomitantly, mRNAs of anti-oxidative enzymes Mn-SOD and glutathion-peroxidase-1 were significantly elevated in whole lung tissue. Interestingly, we found a 1.5-fold decrease of anti-oxidant enzyme hemoxygenase-1 (HO-1) in animals with MCT-treatment and increased blood-flow, versus MCT-treatment only. In addition, mRNA-levels of catalase, another major anti-oxidative player were significantly reduced in both, lungs from the MCT- and from the MCT/PE-group. We tried to localize the increase of the two main pro- and anti-oxidative players NOX-4 and Mn-SOD, respectively, within the diseased lungs. Immunohistochemistry experiments allowed us to literally highlight a cell population, which we had not noticed in the first place:diseased lungs of the MCT/PE, and to a lesser extent of the MCT group displayed an overwhelming influx of large, cytoplasm-rich interstitial NOX-4+ and Mn-SOD+ cells, corresponding to tryptase+ mast cells. In addition to the wide-spread interstitial distribution, NOX-4+ and MN-SOD+ mast cells were observed in the range of pulmonary arteries and bronchioles. In an attempt to reveal a possible mechanism for the massive pulmonary homing of mast cells the recruitment pathway through the SDF-1/CXCR4 couple was positively tested: SDF-1 was expressed by endothelial cells of pulmonary vessels and by perivascular inflammatory cells, while immunofluorescent double-labeling revealed CXCR4-expression by NOX-4+ mast cells. In line with our observation, CXCR4 is known to be expressed by blood-derived mast cells and mast cell chemotaxis has been reported to be mediated by SDF-1 [22,23]. Our group has previously described the implication of CD117+ cells, comprising tryptase-positive mast cells and tryptase-negative bone-marrow derived progenitor cells in human PAH [24], and increase of pulmonary mast cells has been described in human PAH [25], as well as in experimental left-heart independent and left-heart-dependent PH-models [26,27]. Dahal and co-workers have elegantly shown in the monocrotaline rat-model, that mast-cell numbers are significantly increased and that preventive treatment with the specific c-kit inhibitor PLX and the mast-cell stabilizer cromolyn sodium salt (CSS) reduce mast-cell numbers and ameliorate pulmonary vascular remodeling, right-heart hypertrophy and hemodynamics [26]. Interestingly, they were not able to attenuate these three indicators of PH in a therapeutic (non-preventive) fashion. When considering the vascular and peri-vascular localization of oxidative stress markers malondialdehyde and nitrotyrosine (see beneath), one seducing hypothesis appears to be a 'bow and arrow' setting, in which the mast cells (bow) will have produced enough ROS (arrows) to injure their distant target (pulmonary arteries) and trigger a self-supporting pro-oxidative and pro-inflammatory process, which will not be affected by any mast-cell dependant therapy.

Supporting our findings of increased oxidative imbalance in the MCT/PE model, hemodynamic stress induced by increased pulmonary blood flow has been previously proposed as a cause of superoxide anion production by oxidant enzymes, such as NOX-4, in cultured human endothelial cells [18]. Of note, increased ROS production was observed only after cell exposure to pulsed, unidirectional flow, as compared to non-pulsed flow. Differentiation between pulsed and non-pulsed flow in the study of endothelial stress originates from an earlier work by De Keulenaer and co-workers [28], where induction of NOX-4 activity is only observed after exposure of endothelial cell cultures to pulsed oscillatory shear stress, but not to non-pulsed unidirectional shear-stress. Originally, these data support the hypothesis of OS generation in plaque-prone regions of systemic atherosclerotic arteries. It is quite well established, that biomechanical forces exerted on the vessel wall by the flowing blood tend vary at these predilection sites due to oscillations in blood flow [29]. As a consequence, laminar stress is reduced, whereas volume-dependent deformation of the vessel wall is enhanced. In fact, laminar shear stress is believed to protect arteries from developing intimal lesions, such as atherosclerosis, by maintaining endothelial cell nitric oxide synthesis and by enhancing the activity of anti-oxidant enzymes, e.g. HO-1. On the other hand, site-dependent cyclic vessel deformation is known to promote atherosclerosis through an increased formation of ROS and impairment of HO-1 [30,31]. Further more, it has been recently shown, that stimulation of HO-1 induces an improvement of endothelial dysfunction in spontaneously hypertensive Wistar-Kyoto rats by reducing OS and increasing NO availability [32]. In a latest study, Yu and co-workers have shown that ROS overproduction and NOX-4 increase in a trauma/hemorrhage rat model can be prevented and trauma-impaired endothelium-dependent vaso-relaxation restored through stimulation of HO-1 expression [33]. However, our findings also demonstrate deterioration of the oxidative balance through decrease of anti-oxidative enzyme catalase mRNA in both models as compared to controls, and of HO-1 mRNA in the MCT/PE group as compared to MCT alone. This raises the question of a possible mechanism for the downregulation of such protective enzyme-encoding genes. A plausible pathway for this deleterious phenomenon might be triggered by HIF-1 signaling: Loboda and co-workers have recently reported that induction of HIF-1 in human endothelial cells through hypoxia and dimethyloxaloylglycine attenuated the expression of IL-8 and of HO-1 through down-regulation of transcription-factor Nrf2 [34]. The increased expression and important role of HIF-1 in human PAH and experimental PH has been addressed by several authors and is well recognized [35]. It has been reported that experimental pneumonectomy elicits HIF-1 signaling [36]; hence, a decrease of HO-1 and also of catalase would be plausible in our model, especially in the MCT/PE model. Further evidence of a connection between pulmonary arterial remodeling and oxidative/anti-oxidative balance alterations has been recently provided by Podlutsky and co-workers [37]. The investigators tested several major enzymes of the oxidative/anti-oxidative system in F344-rats of different age (3 to 28 month old) in order to obtain an age-related profile on the presence of OS. As aging in the systemic circulation of the elderly is associated with generalized endothelial dysfunction and increased OS probably contributing to increased morbidity and mortality from cardiovascular diseases [38], the rationale of the study was based on observations that pulmonary artery pressure and vascular resistance increase with normal aging in humans. The authors found, that aging in rat pulmonary arteries is associated with impaired acetylcholine-induced relaxation and vascular OS. Amongst others, expression of NOX-4 (mRNA) significantly increased in aged vessels, whereas expression of catalase significantly decreased. In contrast, expression of Mn-SOD, and glutathione peroxidase remained unaltered. Interestingly, this rodent enzymatic aging-profile of pulmonary arteries is congruent to our findings in permanent high flow-challenged monocrotaline treated rats, as compared to controls.

The increasing appearance of pulmonary artery adventitial fibroblasts (PAFB) in the setting of experimental hypoxic PH and their contribution to arterial remodeling has been reported in the past [39]. In the context of human PAH, Li and co-workers have recently studied the expression of different NOX subunits in PAFB of human donors under normoxic and hypoxic conditions, as well as in IPAH-patients [40]. Under hypoxic conditions NOX-4 was significantly upregulated at mRNA and protein levels. Silencing of NOX-4 by siRNA caused reduction of ROS levels under both normoxic and hypoxic conditions and suppressed the significant hypoxic-induced ROS increase. In addition, PAFB proliferation was significantly decreased in cells transfected with NOX-4 siRNA, whereas apoptosis was enhanced. A significant increase of NOX-4 mRNA expression was observed under hypoxic conditions in PAFB from the lungs with IPAH compared to healthy donors.

Further more, the role of NOX-4 in human pulmonary artery smooth muscle cells (HPASMC) has been the subject of a study by Ismail and co-workers [41]. The investigators show that hypoxia induced HPASMC proliferation *in vitro *is accompanied by increased reactive oxygen species generation and NOX-4 gene expression, and is inhibited by antioxidants, the flavoenzyme inhibitor diphenyleneiodonium (DPI), and NOX-4 gene silencing.

Our findings suggest that there may be an accentuated attenuation of the biological anti-oxidant defense system in MCT/PE-rats as compared to controls, but also as compared to monocrotaline challenge only. An imbalance between oxidative and anti-oxidative molecules could contribute to exuberant pulmonary arterial remodeling, since histomorphological and hemodynamic differences between our two PH-models were marked.

### Markers of reactive oxygen species-activity and inflammation are overexpressed in pulmonary arteries of hypertensive rats

To test the hypothesis of direct ROS-involvement in accentuated flow-associated vasculopathy, we studied the expression of malonedialdehyde and nitrotyrosine in all groups by immunohistochemical experiments. These two molecules are established markers of ROS-activity on lipids, proteins and DNA, respectively [42-44]. We found increased production of malonedialdehyde and nitrotyrosine in lung-tissue from MCT- and MCT/PE-animals, as compared to controls. The main sources of both markers were endothelial cells, an observation that has been previously made in lungs from patients with PAH [20]. In addition but to a lesser extent, medial smooth-muscle cells stained for both markers. Of interest, malonedialdehyde was markedly produced by perivascular leucocytes of both pulmonary arteries and veins, suggesting a self-supporting stress circuit through recruited inflammatory cells. This possibility is supported by a recent experimental study from De Miguel and colleagues [45]. The authors report an increased infiltration of T-lymphocytes into the kidney of rats with experimental salt-sensitive arterial hypertension and an increase of NOX-subunits within the attracted lymphocytes associated with an increase of OS-markers in the kidney. These pro-oxidative effects were attenuated by preventive immune-suppression through tacrolimus, resulting in a decrease of lymphocytic infiltrations and eventually decrease of arterial hypertension. This appears of particular interest regarding the close association of inflammatory elements and pro-oxidative activity in our experimental PH-model: as compared to MCT-animals, hypertensive rats with increased blood flow and maximal inflammatory infiltrates within the pulmonary arterial wall exhibited a higher number of remodeled vessels with production of OS markers. Cytokines IL-6, RANTES and fractalkine were overexpressed in MCT/PE and to a lesser extent in MCT, indicating a proinflammatory state within lung-tissue. IL-6 expression was localized to pulmonary arterial endothelium, while the two latter proteins were mostly expressed by perivascular leucocytes in our model.

Our previous articles have highlighted an overproduction of IL-1, IL-6, CCL2 (MCP-1) CXCL1 (fractalkine) and CCL5 (RANTES) [46]. Recently, Furaya et al [47] proposed a hypothetical mechanism leading to pulmonary vascular remodeling via overexpression of IL-6. IL-6 induces proliferation and anti-apoptosis in vascular smooth muscle cells through upregulation of VEGF, and downregulation of BMPR2 and TGFβR2. Upon IL-6 exposure, endothelial cells undergo apoptosis through repressed Tie2 signaling via downregulated Ang-1 expression in smooth muscle cells. Production of CX3CL1 results in recruitment of inflammatory cells, such as lymphocytes and monocytes, which produce enormous amounts of IL-6, while vascular smooth muscle and endothelial cells also produce IL-6 upon stimulation with IL-6. This hypothesis is confirmed experimentally as interleukin-6 overexpression induces pulmonary hypertension in mice [48]. All the inflammatory mediators we have shown to be overproduced in PAH are linked to this key cytokine. Indeed, in mouse serum, mouse CCR2 protein (the receptor for CCL2) is necessary for expression of mouse IL6 protein that is increased by experimentally induced sepsis in mouse [49], and IL1 protein increases expression of CCL2 protein [50]. IL-1 also increased directly IL-6 mRNA levels by a protein kinase C-independent mechanism [51] and IL-1 receptor antagonist treatment reduces pulmonary hypertension generated in rats by monocrotaline [52]. Moreover, IL-1 may impair the therapeutic effects of prostacyclin analogues such as iloprost and carbaprostacyclin by attenuating cyclic AMP production by human pulmonary artery smooth muscle cells in response to these drugs [53]. At last, in dendritic cells, mouse CCL5 protein increases expression of mouse IL6 mRNA [54].

Further more and in accordance with previous reports [55], we found decreased expression of PH-associated genes BMPR2 and of BMPR1A in both, MCT and MCT/PE, underlining the molecular similarities of those two animal-models with human familial or idiopathic PAH when it comes to alterations of BMP/TGF-β signaling.

### Increased blood flow leads to modified arterial wall proportions in MCT-induced PH

The combination of MCT and pneumonectomy in young rats as a model for pulmonary hypertension has been studied for the first time by Tanaka and colleagues [56]. The authors describe excessive intimal and medial remodeling of distal pulmonary arteries in those animals, as compared to MCT alone, which is nearly absent in animals undergoing pneumonectomy alone. In a more recent study, Homma and co-workers utilized a MCT-penumonectomy model in order to test preventive and curative effects of dehydroepiandrosterone in experimental pulmonary hypertension [57]. One week after MCT injection, left pneumonectomy was performed and sacrifice was done 4.5 weeks later (day 40). PH was accompanied by severe pulmonary vascular remodeling, consisting of small pulmonary arterial medial wall thickening, increased adventitial cellularity and to a lesser extent arteriolar neointimal lesions.

Indeed, microscopic morphometrical evaluation revealed significant differences between our two pulmonary hypertensive groups. First, excessive muscularization of arterioles of less than 50 μm in external diameter was observed in both, MCT- and MCT/PE-animals. However, this phenomenon was quantitatively more pronounced in the latter group. In addition to arteriolar muscularization, the MCT/PE group displayed discrete intimal thickening with increased cellularity in small arterioles, as it has been described by Tanaka and by Homma. This observation might be of importance in the context of an experimental PH-model, since most PH-models generate medial thickening and adventitial remodeling, but lack intimal lesions, a hallmark of human PH.

Our results are Further more consistent with recent data showing the consequences of right-sided lobectomies in a rat model indicating that arteriolar muscularization occurs in rats with triple-lobectomy (quasi equal to pneumonectomy), but not in double-lobectomy [58]. The authors describe a muscular thickening of pulmonary arterioles in between an external diameter of 30 to 80 μm, while remodeling of pulmonary arteries of over 100 μm is not reported. Interestingly, our study reveals qualitative differences of arterial remodeling between the MCT- and the MCT/PE-group, concerning pulmonary arteries with an external diameter ranging from 101 to 450 μm. Medial and adventitial thickening in MCT/PE-animals exceeded values in MCT-treated rats significantly. In fact, exuberant adventitial cellularity was observed to a smaller extent in MCT, while consistently present in MCT-PE. In accordance with excessive wall dimensions, MCT/PE exhibited significantly lower surface areas of arterial lumina than MCT, underlining the obstructive character of vascular remodeling.

### Dendritic cells and CD68^+ ^cells are massively recruited to remodeled arteries of hypertensive rats with increased blood-flow

We have previously demonstrated that DCs may be involved in hypertensive pulmonary vasculopathy, since they are recruited to arterial lesions in experimental and human PH. DCs are mainly known as antigen presenting cells connecting the innate immunity to the adaptive immune response when facing danger signals and inducing tolerance to self-antigens in a non alerted immunitary state [59]. Under inflammatory conditions, DCs orchestrate the immune response through activation and initialization of T-lymphocytes, but may also communicate with B-cells and fibroblasts [60]. In previous reports, DCs have been demonstrated to organize arterial inflammatory lesions in systemic atherosclerosis [61]. Noteworthy, a recent study has demonstrated an increased DC resistance towards organic and hydrogen peroxides through overproduction of manganese superoxide dismutase [62]. In systemic arteries DCs accumulate in regions displaying hemodynamic stress by turbulent flow conditions and being prone to hypertensive intimal lesions [17]. We observed intense recruitment of DCs into the wall of affected pulmonary arteries in MCT/PE-rats and to a lesser extent in MCT-rats. DCs were uniformly present within the dense adventitial cell-accumulations in MCT/PE and frequently infiltrated medial smooth-muscle-cells, up to subintimal regions. Noteworthy, in all groups, including control animals, numerous DCs were detected within BALT, while interstitial DCs were sparse. This finding is supported by previous observations mainly locating myeloid dendritic cells within the range of isolated pulmonary lymphoid follicles and BALT in the context of pulmonary interstitial diseases, in particular rheumatoid arthritis (RA) and Sjögren's syndrome (SS) [63,64].

In MCT/PE-animals, adventitial thickening mainly consisted in cells staining for CD68. However, in arteries presenting intense adventitial cellularity, we detected a pronounced medial infiltration by CD68^+^/vimentin^+ ^cells. These cells, to some extent, morphologically displayed a fusiform pattern and were uniformly arranged within the muscular compartment, explaining difficult differentiation on HE-stained slides. Frid and co-workers have reported the arterial recruitment of mesenchymal precursors of a monocyte/macrophage lineage, including circulating fibrocytes, in hypoxic lungs of calves and rats [65]. Recently, Burke and colleagues have reported influx of immune cells, including dendritic cells, into the pulmonary arterial walls of Wistar-Kyoto rats during sustained hypoxia, and concomitant increase of pro-inflammatory cytokines, growth factors and adhesion molecules [66]. More specifically, the investigators observed an increase in the appearance of OX62+ dendritic cells in the peri-adventitial region of vessels from chronically hypoxic animals on days 7 and 28. Interestingly, it has also been shown that activated macrophages express vimentin only in proinflammatory conditions under the influence of proinflammatory cytokines and that macrophagic vimentin secretion is an important source of oxidative metabolite generation [67]. These features would support a self-supporting concept of inflammatory action/reaction and OS.

## Conclusion

We report increased OS activity in hypertensive lung vasculature of MCT-treated, high-flow challenged rats associated with an imbalance of pro- and anti-oxidative enzymes and massive ROS-generating mast-cell-influx. MCT/PE-animals display severe intimal, medial and adventitial, functionally relevant remodeling with pro-inflammatory cytokine-dependant massive infiltration of CD68^+^/vimentin^+ ^cells and immune-modulating dendritic cells into the pulmonary arterial wall. Our data indicate that, in experimental PH, mast cell-steered increase of OS and peri-vascular inflammatory cell-signaling are related and partly induced by increased blood-flow and could favor pulmonary arterial remodeling.

## List of abbreviations

O2^-^: superoxide; ALK: activin receptor-like kinase; BALT: bronchiolar-associated lymphatic tissue; BMPR: bone morphogenetic protein receptor; DC: dendritic cell; HIF: hypoxia inducible factor; HO-1: heme oxygenase-1; HPASMC: human pulmonary artery smooth muscle cells; IL-6: interleukin-6; IPAH: idiopathic pulmonary arterial hypertension; LV: left ventricle; MCT/PE: monocrotaline/pneumonectomy; MCT: monocrotaline; Mn-SOD: manganese-superoxidismutase; mPAP: mean pulmonary artery pressure; NIH: National Institutes of Health; NO: nitric oxide; NOX-4: NADPH oxidase-4; OS: oxidative stress; PA: pulmonary artery; PAFB: pulmonary artery adventitial fibroblasts; PAH: pulmonary arterial hypertension; PBS: phosphate buffered saline; PDGF: platelet-derived growth-factor; PEEP: positive end-expiratory pressure; PH: pulmonary hypertension; RA: rheumatoid arthritis; ROS: reactive oxygen species; RT-PCR: real time-polymerase chain reaction; RV: right ventricle; RVSP: right ventricular systolic pressure; S: septum; SS: Sjögren's syndrome

## Competing interests

The authors declare that they have no competing interests.

## Authors' contributions

PD and FP drafted the manuscript. PD and FP carried out animal experiments and RT-PCR and designed the study. PD, IDG and NR carried out histochemistry, immunohistochemistry and immunofluorescence assays. PD (with the participation of FC) carried out morphometry and histomorphological analysis. MCC, DM and MH participated in the design of the study and performed the statistical analysis. MG, EF, OM, and GS helped to coordinate the study. All authors read and approved the final manuscript.

## Authors' information

PD is working as a pathologist in Hôpital Marie Lannelongue, Le Plessis Robinson, housing the research unit 999 of the 'Institut National de la Santé et de la Recherche Médicale' which focuses on fundamental research on the pathophysiology of pulmonary hypertension and is directed by MH. He works also as a consultant to the French Referral Center on Pulmonary Hypertension which is located in Antoine-Béclère Hospital, in Clamart.
